# The Role of Inflammation in the Pathogenesis of Viral Respiratory Infections

**DOI:** 10.3390/microorganisms12122526

**Published:** 2024-12-07

**Authors:** Arnaud John Kombe Kombe, Leila Fotoohabadi, Yulia Gerasimova, Ravikanth Nanduri, Pratik Lama Tamang, Monisha Kandala, Theodoros Kelesidis

**Affiliations:** Division of Infectious Diseases and Geographic Medicine, Department of Internal Medicine and Infectious Diseases, University of Texas Southwestern Medical Center, Dallas, TX 75390, USA

**Keywords:** viral respiratory infection, inflammation, influenza virus, SARS-CoV-2, respiratory syncytial virus (RSV)

## Abstract

Viral respiratory infections (VRIs) are a leading cause of morbidity and mortality worldwide, making them a significant public health concern. During infection, respiratory viruses, including Influenza virus, SARS-CoV-2, and respiratory syncytial virus (RSV), trigger an antiviral immune response, specifically boosting the inflammatory response that plays a critical role in their pathogenesis. The inflammatory response induced by respiratory viruses can be a double-edged sword since it can be initially induced to be antiviral and protective/reparative from virus-induced injuries. Still, it can also be detrimental to host cells and tissues. However, the mechanisms that differentiate the complex crosstalk between favorable host inflammatory responses and harmful inflammatory responses are poorly understood. This review explores the complex interplay between viral pathogens and the host immune response, mainly focusing on the role of inflammation in the pathogenesis of VRIs. We discuss how inflammation can both contain and exacerbate the progression of viral infections, highlighting potential therapeutic targets and emerging drugs for modulating the aberrant inflammatory responses during VRIs.

## 1. Introduction

Acute viral respiratory infections (VRIs) cause a significant death rate worldwide, either alone or in the presence of co-infection with secondary bacterial infections [1,2,3]. Most viral respiratory infections are relatively mild, uneventful, and self-limiting. However, severe lower viral respiratory infections, including bronchiolitis, croup, pneumonia, exacerbation of asthma, or chronic obstructive pulmonary disease (COPD), can lead to respiratory failure, morbidity, mortality, and cardiovascular or neurological sequelae. The severity of the infection largely depends on the type of the virus involved and the host’s ability to mount protective immune responses [4,5]. Amongst the pathogens causing RVIs, respiratory viruses, including influenza viruses (IVs), human parainfluenza viruses (hPIVs), respiratory syncytial virus (RSV), human metapneumovirus (hMPV), human rhinovirus (HRV), and human coronaviruses (hCoVs—SARS-CoV-2), are the most frequent agents implicated in the etiology of these infections. Despite considerable morbidity and mortality associated with RVIs, there are limited antiviral treatments and a limited understanding of the mechanisms underlying the pathogenesis of these infections. One increasingly recognized factor that may contribute to the pathogenesis of viral respiratory infections leading to severe lower RVIs is aberrant host inflammatory response to virus infection, which in some cases can manifest as a cytokine storm [6,7,8,9]. Epidemiological data have suggested that increased inflammation during RVIs is associated with worsening retained lung function after each episode [10,11].

Inflammation is a complex response in the host’s defense mechanism against viral infections and associated tissue homeostasis and repair [12,13]. Uncontrolled or improperly regulated inflammatory response can lead to tissue damage and severe lung pathology [14,15,16]. Therefore, understanding the balance between protective and detrimental effects of inflammation is crucial for developing therapeutic strategies.

In this review, we highlight the role of inflammation in the pathogenesis of RVIs. We discuss distinct types of primary main inflammatory responses and their implications for treatment or prevention strategies.

## 2. Inflammation as a Double-Edged Sword in Viral Infections

Inflammation is a vital early response in viral respiratory infections, with type I interferons (IFNs) playing a crucial role in viral clearance and the activation of innate and adaptive immune responses [17,18]. While beneficial for controlling viral replication, excessive inflammation can lead to detrimental outcomes. Overproduction of pro-inflammatory cytokines, particularly IFN-γ, has been linked to exacerbations of conditions like chronic obstructive pulmonary disease (COPD) and severe pneumonia [19]. For example, higher levels of IFN-γ have been observed in the lungs of patients who succumbed to infections compared to survivors [20,21]. This suggests that while inflammation is necessary for fighting infections, an uncontrolled inflammatory response can cause extensive tissue damage due to reactive oxygen species (ROS) and proteolytic enzymes, potentially resulting in acute respiratory distress syndrome (ARDS) and fatal pneumonia.

### 2.1. Protective Role of Inflammation

**Figure 1:**
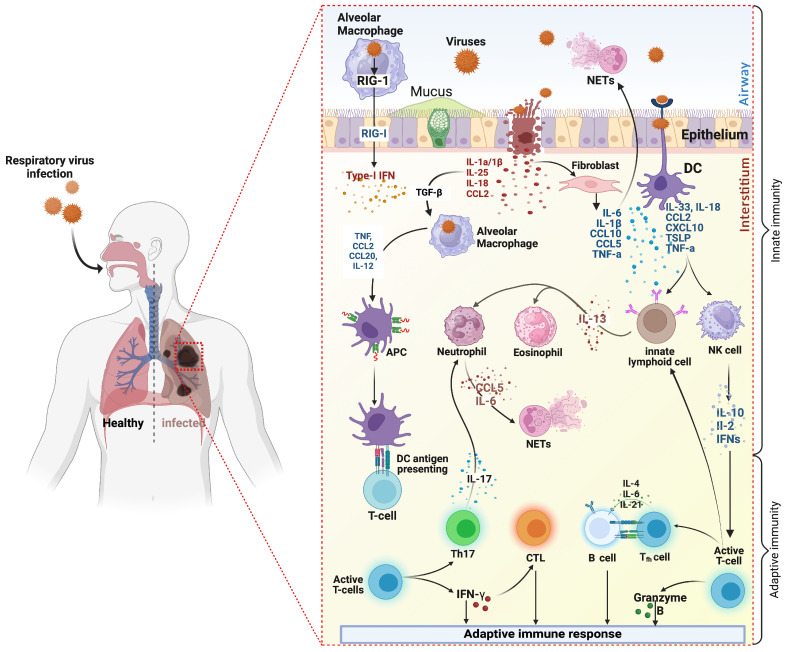
Immune Response to Respiratory Viral Infections. Upon infection of human airway epithelia cells by respiratory viruses, viruses are first detected by innate endosomal and cytoplasmic sensors in infected epithelial and stimulated dendritic cells (DCs). These cells then secrete type I and type III interferons (IFNs) through activation of retinoic acid-inducible gene I (RIG-I) and production of pro-inflammatory cytokines such as interleukin-1β (IL-1β) and IL-18, and chemokines including CC-chemokine ligand 2 (CCL2). The secreted immune mediators activate adjacent cells, including fibroblasts, epithelial cells, and neighboring innate immune cells (CDs), which, in turn, release more chemokines (CCL2, CCL20, CXCL10, and TSLP) and cytokines (IL-12 and tumor necrosis factor (TNF)-α, IL-6, etc.)) that subsequently boost activation and recruitment of NK-cells and neutrophils at the site of the infection, as well as activation of macrophage through the growth factor-β (TGF-β). Maturation of tissue-resident DCs occurs alongside antigen acquisition and activation of immature antigen-bearing respiratory DCs, which results in their mobilization and migration to initiate adaptive immune responses. T-cells and NK-cells are crucial in releasing IL-17 and IFN-γ to stimulate an antiviral response or support the granzyme B-mediated elimination of virus-infected cells. On the other hand, B cells generate antibodies that specifically target viral antigens to offer adaptive immune protection for the host. Excessive production of cytokines and chemokines during infection is prone to uncontrolled inflammatory response, leading to inflammatory diseases. (Created with BioRender.com, 2024, accessed on 6 October 2024).

Inflammation is essential for restoring homeostasis following viral infections. This process begins with destroying damaged host cells and removing inflammatory mediators, which are crucial for resolving infections. Soluble antimicrobial substances like defensins and lysozyme are released during inflammation, enhancing host defense [22,23]. Additionally, the complement system (a cascade of serum protein interactions) mediates opsonization and directly targets pathogens by recognizing pathogen-associated molecular patterns (PAMPs). Activation of the complement system can also occur via antibodies from adaptive immunity, facilitating adequate pathogen clearance [24,25]. Surfactant proteins A and D further bridge innate and adaptive immunity by binding to antigen-presenting cells, enhancing antigen uptake and presentation [26]. Inflammation recruits dendritic cells to activate the adaptive immune response and mobilizes macrophages and neutrophils to the infection site, producing cytokines and chemokines that enhance immune cell infiltration. For instance, in immunocompetent individuals, this type 1 inflammatory response promotes adequate clearance of respiratory viral infections while resolving inflammation post-clearance (Figure 1) [27,28]. Detailed mechanisms of resolving post-infection inflammation is discussed hereinafter.

### 2.2. Pathological Consequences of Excessive Inflammation

While inflammation aids recovery, excessive inflammatory responses during viral respiratory infections can lead to significant airway pathology. Severe inflammation results in immune-mediated damage to the lung and vascular endothelium, heightening the risk of complications such as diffuse alveolar damage, bacterial pneumonia, and ARDS [7]. For example, both influenza A virus (IAV) and RSV have evolved mechanisms to inhibit IFN production, allowing them to circumvent immune defenses. Although a robust IFN response is essential early in infection to limit viral replication, prolonged production can impede epithelial cell repair and disrupt macrophage antibacterial functions. Moreover, IAV infection can skew macrophage polarization towards a pro-inflammatory state, exacerbating cytokine release [29]. In individuals with compromised respiratory health, such as asthmatics, inflammatory responses may be dysregulated, leading to persistent viral replication and chronic inflammation. This sustained inflammation contributes to chronic airway diseases, such as asthma and COPD, worsening conditions upon RVIs [30,31]. In severe cases, aberrant inflammasome activation can trigger a cytokine storm, leading to extensive damage in pulmonary tissues. For instance, the dysregulated activation of NLRP3 in SARS-CoV-2 infection has been linked to severe lung inflammation and tissue damage [32,33]. This illustrates the necessity for a carefully regulated inflammatory response to facilitate viral clearance while preventing tissue destruction. Understanding these dynamics is crucial for developing effective therapies to manage viral infections and mitigate their pathological consequences.

## 3. Main Mediators of Inflammation in Viral Respiratory Infections

Several studies reported the role of inflammation in the lungs of patients with RVIs. Pro- and anti-inflammatory mediators such as cytokines, chemokines, bioactive lipids and antiproteases have been reported to play a crucial role in the pathogenesis of RVIs and lung inflammation [34]. Especially, cytokines and chemokines, produced in the majority by airway epithelial cells and tissue-resident alveolar macrophages, are the primary mediators found to direct immune cell activity and movement towards the infection site and aid in the proliferation, maturation, and activation of immune cells, playing a crucial role in the host’s inflammatory response (Figure 1) [35,36].

### 3.1. Cytokines

Cytokines are small signaling glycoproteins that regulate inflammatory immune responses during RVIs and operate within a complex interplay between viral pathogens and infected airway cells. Viral pathogen-associated molecular patterns (PAMPs) are recognized by pattern recognition receptors (PRRs), such as toll-like receptors (TLRs), which trigger the expression of various cytokines. Cytokines bind to cell surface receptors, prompting target cells to express specific genes. Cytokines are classified into four structural families: the four alpha-helix bundle family (including the IL-2, IFN, and IL-10 subfamilies), the IL-1 family, the IL-17 family, and the cysteine-knot cytokines (TGF-β superfamily), and further categorize into various types, including interferons, colony-stimulating factors (CSF), interleukins, transforming growth factors (TGF), and tumor necrosis factor (TNF), which are secreted through paracrine, autocrine, and endocrine pathways [37]. These molecules are linked to pro-inflammatory and anti-inflammatory responses in infections and immune disorders.

Cytokines play a crucial role in the immune response to RVIs, mainly through the actions of type I and type III IFNs and other pro-inflammatory cytokines. Type I IFNs (IFN-α and IFN-β) are produced in response to viral detection and activate the JAK/STAT and NF-κB signaling pathway via their receptor, IFNAR. This response induces a wide array of interferon-stimulated genes (ISGs) that limit viral replication and spread while enhancing immune cell functions such as phagocytosis and activating dendritic cells (DCs). Type I IFNs facilitate the adaptive immune response by promoting DC maturation and antigen presentation, activating T cells, and stimulating antibody production. Type III IFNs (IFN-λ) have a similar antiviral profile but are primarily produced by airway epithelial cells, making them particularly effective in respiratory infections [38]. Other cytokines like IL-1, IL-18, IL-6, and TNF-α modulate immune responses by promoting inflammation and influencing immune cell activity. IL-6 aids the transition from innate to adaptive immunity, while TNF-α enhances cytotoxic activity and impairs viral replication. Granulocyte colony-stimulating factor (G-CSF) and granulocyte-macrophage colony-stimulating factor (GM-CSF) further support immune responses by promoting the differentiation and activation of myeloid cells, essential for adequate viral clearance. These cytokines orchestrate a multifaceted immune reaction, balancing antiviral activity and inflammation to combat viral infections [38,39,40].

### 3.2. Chemokines

Like cytokines, chemokines are crucial mediators secreted by airway epithelial cells and pulmonary macrophages for orchestrating immune responses during viral infections, especially in the respiratory system. Their primary role is recruiting innate and adaptive immune cells to the lung and other infected airway tissues to amplify the immune response and release cytotoxic and inflammatory factors [41]. Key chemokines, like CCL2, CXCL8 (IL-8) and CXCL10 (IP-10), guide the movement of immune cells to sites of inflammation. For instance, CXCL8 recruits neutrophils, enhancing their activity to fight infections. Still, excessive levels can lead to ARDS due to tissue damage from neutrophil proteases and reactive oxygen species (ROS) [38]. Additionally, IL-8 may contribute to eosinophil aggregation in asthma, though the mechanisms are not fully understood [19,37,38,40]. IP-10 plays a multifaceted role by promoting the migration and activation of T cells, monocytes, and NK cells, helping regulate immune responses. Elevated IP-10 levels are linked to chronic inflammation, indicating its potential as a therapeutic target [38]. Moreover, CCL5 (RANTES), another type of chemokines, recruits T cells and eosinophils, activating them for effective immune responses. However, its effects can vary; while CCL5 can exacerbate inflammation in respiratory syncytial virus (RSV) infections, its absence may reduce survival in influenza cases [34,38,40]. While chemokines are vital for mounting effective immune responses, their excessive production can lead to heightened inflammation and tissue damage. This delicate balance highlights the need to understand chemokine dynamics for developing therapeutic strategies that effectively combat viral infections while minimizing harmful inflammatory responses and preserving epithelial integrity [34,38,40].

### 3.3. Other Inflammation-Secreted Mediators

In addition to cytokines and chemokines, other secreted inflammatory mediators such as prostaglandins (PGs), leukotrienes (LTs), histamines, and proteases/antiproteases play significant roles in the pathogenesis of inflammation during RVIs. In the course of infection, prostaglandins and leukotrienes exert complex and sometimes opposing effects depending on their concentration, receptor interactions, and the phase of inflammation.

Prostaglandins, derived from arachidonic acid, are produced by various cell types, including airway epithelial cells and macrophages [42]. PGE2 and prostacyclin (PGI2) are particularly the main inducers of hallmark signs of inflammation and the most involved in the pathogenesis of RVIs-associated inflammatory response. They contribute to virus-induced inflammation, pain, and fever by promoting vasodilation, increasing vascular permeability, and enhancing immune cells’ recruitment into infection sites [43]. PGE2 also enhances the production of pro-inflammatory cytokines in RVIs, including IL-1β and TNF-α, amplifying the immune response. However, prostaglandins can either exacerbate or resolve inflammation depending on their concentrations and the type of prostaglandin receptor engaged. For instance, prostaglandin J2 metabolites act later in inflammation to exert anti-inflammatory effects induced by viruses (bacteria as well), reducing tissue damage and promoting the resolution of the inflammatory process [44,45]. Besides, in RSV infection, virus replication correlates with the epithelial cell production of pro-inflammatory PGE2, which resolves upon virus clearance [44].

Similarly, leukotrienes derive from arachidonic acid and are primarily produced in the respiratory tract by mast cells, eosinophils, and alveolar macrophages upon stimulation through RVIs such as SARS-CoV-2 infection [42]. They are potent mediators that induce bronchoconstriction and enhance mucus production, exacerbating respiratory symptoms [46]. For instance, LTB4 and cysteinyl leukotrienes (LTC4, LTD4, LTE4), play significant roles, primarily in the recruitment and activation of leukocytes like neutrophils and eosinophils to the site of injury. LTB4, a potent chemoattractant, increases leukocyte infiltration, whereas cysteinyl leukotrienes, in synergy with prostaglandins, contribute to increased vascular permeability, mucus secretion, and smooth muscle contraction, particularly in respiratory conditions like asthma. In influenza A viral infection, leukotrienes, particularly LTB4, can cause disease tolerance to influenza infection [44,45,47], and reduce SARS-CoV-2-associated severity in COVID-19 patients [48]. However, the production of leukotrienes can lead to further tissue damage if produced in excess [47,49]. Overall, in respiratory virus-induced inflammation, such as during viral infections like the flu or RSV, the excessive production of prostaglandins and leukotrienes contributes to the pathophysiology, causing airway obstruction, bronchoconstriction, and mucus overproduction. Inhibiting these mediators, particularly through cyclooxygenase (COX) or 5-lipoxygenase inhibitors, has been explored as a therapeutic strategy for managing inflammatory diseases like asthma and COPD [50].

Antiproteases, such as alpha-1 antitrypsin and secretory leukocyte protease inhibitors, are produced to counterbalance the effects of proteolytic enzymes released during inflammation. These proteins help protect lung tissue from damage caused by inflammatory cells and respiratory viruses such as HRV [51]. However, the balance between proteases and antiproteases can be disrupted in RVIs, leading to tissue injury and impaired lung function. These mediators create a complex inflammatory milieu that can contribute to viral clearance or exacerbate tissue damage, highlighting their dual roles in respiratory infections.

### 3.4. Pro-Resolving Mediators

The host possesses a specific resolving mechanism that tracks and restores the homeostasis balance post-infection. After the clearance of respiratory infections like HRV, RSV, and SARS-CoV-2, the host resolves inflammation through a coordinated process involving specialized pro-resolving mediators (SPMs), regulatory T cells (Tregs), and anti-inflammatory cytokines like IL-10. SPMs, derived from omega-3 fatty acids like EPA and DHA, play a key role in returning the respiratory system to homeostasis. After infection, these lipid mediators promote the resolution of inflammation by clearing pro-inflammatory cytokines, enhancing efferocytosis (the removal of dead cells), and limiting immune cell activation (reviewed in [52]). For example, during HRV infection, resolvin D1 (RvD1) promotes neutrophil apoptosis and enhances macrophage-mediated clearance of apoptotic cells, which helps stop inflammation and repair tissue. Similarly, in RSV infections, SPMs like protectin D1 (PD1) and maresin 1 (MaR1) aid in controlling inflammation, promoting epithelial regeneration, and facilitating tissue repair while also preventing excess immune cell activation and lung damage [44,52].

In the case of SARS-CoV-2, SPMs such as RvD1 and RvD2 help mitigate the cytokine storm, a key feature of severe COVID-19 [52,53]. These mediators reduce the production of pro-inflammatory cytokines, decrease immune cell activation, and promote the clearance of inflammatory cells from the lungs. For instance, RvD1 suppresses neutrophil recruitment and reduces macrophage activation, thus preventing tissue injury. RvD1 also enhances the capacity of alveolar macrophages to clear pathogens and apoptotic cells, promoting tissue healing and reducing inflammation. Similarly, as Influenza virus infections can significantly reduce alveolar macrophage populations and reprogram them toward a pro-inflammatory state, by leveraging endogenous macrophage resolution pathways with exogenous cys-maresins (which enhance host defense by targeting lung macrophages), the host can bolster macrophage resilience post-influenza infection [52].

T-regs are crucial for regulating immune responses and play a pivotal role in inflammation resolution by producing anti-inflammatory cytokines like IL-10. After infection, Tregs help suppress overactive immune responses and promote the transition from inflammation to repair [44,52,53,54]. In RSV and SARS-CoV-2 infections, Tregs produce IL-10, which limits the release of pro-inflammatory cytokines such as TNF-α and IFN-γ, thereby reducing tissue damage. IL-10 signaling is crucial for preventing chronic inflammation and supporting tissue regeneration. Tregs also help restore lung homeostasis by modulating the immune response, reducing immune cell activation, and promoting tissue repair mechanisms [44,52,54].

In HRV infection, Tregs also help resolve inflammation by enhancing IL-10 production, attenuating the airways’ inflammatory response. This regulatory mechanism is vital for preventing chronic conditions like asthma, where unresolved inflammation can lead to airway hyperresponsiveness and fibrosis. Additionally, SPMs like RvD1 and MaR1 work synergistically with Tregs to promote IL-10 production, further supporting the resolution of inflammation and tissue healing [52,53].

Together, SPMs, Tregs, and IL-10 form an integrated network that resolves inflammation and promotes tissue repair after infection. These mechanisms are crucial for preventing chronic inflammation and protecting the lungs from long-term damage. Infections like HRV, RSV, and SARS-CoV-2 trigger a complex immune response that, when properly regulated, can effectively resolve inflammation. Harnessing these endogenous resolution pathways could provide therapeutic opportunities for treating respiratory infections and chronic inflammatory diseases by restoring lung function and preventing ongoing tissue damage.

## 4. Host Cells Involved in Respiratory Virus-Induced Inflammatory Response

The lung develops various defense mechanisms, including structural, mechanical, and enhanced mucociliary clearance, supported by a robust, effective immune response. This host inflammatory response, characterized by the mediators mentioned above, involves several host tissue cells activated upon viral infection and includes non-immune cells such as epithelial and endothelial cells, and innate immune cells such as monocytes/macrophages, dendritic cells, neutrophils, natural killer (NK) cells, followed by adaptive immune cells, including T and B cells. The crucial role played by these cells in the immune response against RVIs mainly includes the secretion of inflammatory mediators, such as cytokines, chemokines, prostaglandins, leukotrienes, and antiproteases.

### 4.1. Inflammatory Responses Induced by Epithelial Cells

Airway epithelial cells play a crucial role in inflammation induced by respiratory viruses by secreting various cytokines, chemokines, antimicrobial peptides, and other factors. Upon active respiratory viral infection, viral particles infiltrate the upper and subsequently spread across the lower airway epithelial cells, where they replicate and trigger the host’s innate and adaptive immune responses (Figure 1). It has been shown that infections with HRV, RSV, or hPIVs result in significant pyroptosis-related respiratory epithelium damage, characterized by an increased secretion of pro-inflammatory molecules, including cytokines (IL-1, TNF-α, GM-CSF, IL-6, IL-11) and chemokines (RANTES, IL-8, MIP-1α) [55,56,57]. Similarly, evidence has shown that infection with SARS-CoV-2 is associated with epithelial cell inflammation, of which exacerbation is considered a central driving cause of lung tissue damage and COVID-19 severity [58,59,60,61]. Key cytokines like IL-6 and TNF-α are released in response to RVIs, such as influenza and RSV, promoting immune cell recruitment and modulating the immune response. As previously described, IL-6 facilitates the transition from innate to adaptive immunity, while TNF-α enhances cytotoxic activity and inhibits viral replication. Chemokines such as IL-8 recruit neutrophils, while IP-10 and CCL5 attract T cells and monocytes, influencing inflammation and immune responses during infections like COVID-19. Additionally, epithelial cells secrete antimicrobial agents, such as lactoferrin and β-defensins, which directly inhibit pathogens, protect against tissue damage, and support wound healing, underscoring their dual role in immunity and inflammation [38,39,40].

### 4.2. Inflammatory Responses Induced by Endothelial Cells

Endothelial cells, while not the primary targets of most respiratory viruses, can still be infected, with the impact depending on the specific virus involved. For instance, RSV infects pulmonary endothelial cells, leading to cell activation and death [62]. In contrast, influenza viruses typically do not infect human endothelium but can replicate in avian endothelial cells. Indeed, while the IV infection occurs in respiratory epithelial cells, the adjacent endothelial cells are responsible for mounting the pro-inflammatory response [63], which then induces damage to epithelial cells and the epithelial-endothelial tight junctions [56]. Infections that target endothelial cells can exacerbate lung injury by increasing vascular permeability and edema, as seen in hantavirus pulmonary syndrome, which causes severe microvascular leakage and has a mortality rate of 38% [64].

Endothelial cells also play a critical role in modulating the immune response by facilitating the migration of leukocytes into the lungs [64]. Following infection, pro-inflammatory cytokines and growth factors released from surrounding tissues enhance endothelial permeability and promote leukocyte extravasation. Activated endothelial cells secrete additional cytokines such as IL-6 and TNF-α, which can worsen lung damage if produced in excess [38,64]. These uncontrolled processes may lead to a “cytokine storm,” exacerbating respiratory distress. Thus, endothelial cell involvement is crucial for understanding pulmonary injury during RVIs, balancing the need for an effective immune response with the risk of excessive inflammation.

### 4.3. Inflammatory Responses Induced by Macrophages

Alveolar macrophages, the lungs’ resident immune cells, are crucial in responding to RVIs. Typically kept in a suppressive state, they shift to a pro-inflammatory phenotype upon viral exposure, enabling them to initiate immune responses. For instance, during RSV infection, these macrophages produce early cytokines and interferons essential for antiviral defense. Their role is vital in orchestrating immune responses, clearing viral pathogens from the lungs, and balancing inflammation and protection [38]. Specifically, the host releases macrophages, which produce antiviral factors, cytokines, and chemokines that alter local airway niche [65] and activate immune and non-immune cell inflammation. Notably, and as reviewed by Tan et al. [66], the infected host epithelial cells release type I (IFNα/β) and type III (IFNλ) interferons, interleukins (IL)-6, IL-8, IL-12, RANTES, macrophages-associated inflammatory protein 1α (MIP-1α) and monocytes-associated chemotactic protein 1 (MCP-1), which induce innate immune cell infiltration, responsible for the production of type II interferon (IFNγ), IL-2, IL-4, IL-5, IL-9, and IL-12. Thus, macrophages are crucial regulators of inflammatory responses during RVIs.

### 4.4. Inflammatory Responses Induced by Other Immune Cells

Lymphocytes play a crucial role in the inflammatory response to respiratory viruses, such as influenza, RSV, and SARS-CoV-2. Innate Lymphocytes, including NK cells, are critical players in the early response to viral infections. They produce IFN-γ, which aids in clearing virus-infected cells. NK cells can recognize infected cells through specific receptors, eliminating them via various mechanisms, including cytotoxic granules and antibody-dependent cell-mediated cytotoxicity (ADCC). However, some viruses, like influenza, can evade NK cell activity through inhibitory signaling [38]. Furthermore, unconventional T Cells such as natural killer T (NKT) cells and γδ T cells contribute to the immune response. iNKT cells enhance CTL and NK cell responses while limiting lung damage, especially during infections with RSV and influenza. Conversely, γδ T cells can either suppress or exacerbate inflammation by inducing secretory cytokines (IL-1, IL-6, IFN-α, and IFN-β), depending on the viral context [38]. Furthermore, DCs act as critical orchestrators, activating T cells and producing pro-inflammatory cytokines (Figure 1). Different subsets, including conventional DCs and plasmacytoid DCs (pDCs), respond variably depending on the virus. For instance, pDCs can suppress inflammation during RSV infection. Even though lymphocytes are essential for viral clearance, their activities can lead to lung injury if not appropriately regulated. Thus, depending on the specific type of RVI, the complex crosstalk between immune cells such as NK cells, DCs, T cells, γδ T cells may differentially regulate the inflammatory milieu.

## 5. Key Pathways in Inflammatory Response upon Respiratory Viral Infection

### 5.1. NF-κB Activation Pathway

The NF-κB pathway is integral to the host’s inflammatory response during viral infections (Table 1). Viral components, such as double-stranded RNA from viruses like influenza, can activate this pathway via pattern recognition receptors (PRRs), particularly Toll-like receptors (TLRs). TLRs recruit adaptor proteins like MyD88 upon activation, activating the IκB kinase (IKK) complex. This results in the phosphorylation and degradation of IκB proteins, freeing NF-κB dimers, primarily p65/p50, to translocate to the nucleus [37]. There, they induce the transcription of pro-inflammatory cytokines such as TNF-α, IL-6, and IL-12. This cytokine release not only recruits immune cells but also enhances local inflammation [37,67,68,69].

**Figure 2:**
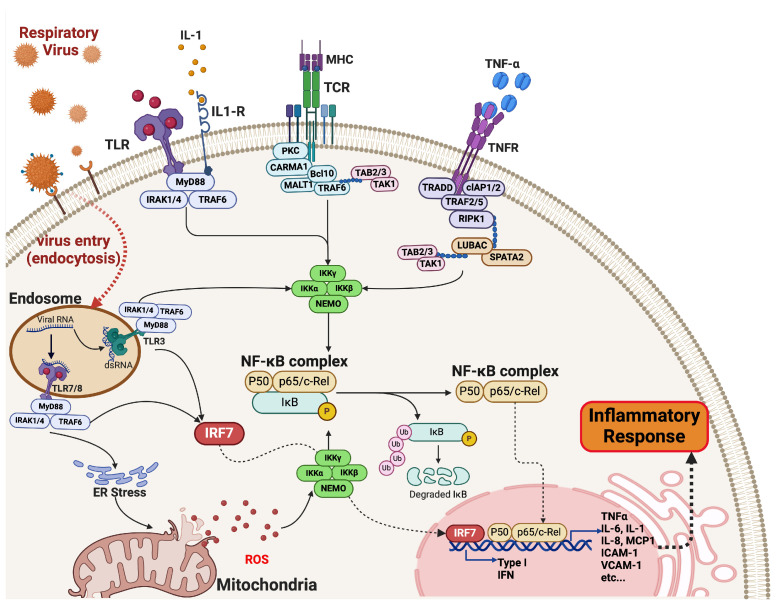
Activation of the NF-κB Pathway during Respiratory Viral Infections. Upon infection, respiratory viruses enter epithelial cells through the endocytosis pathway by a specific cell-receptor binding. Within the endosomes, while viral single-stranded RNA (ssRNA) activates the toll-like receptors (TLR)-7 and TLR8, the intermediate double-stranded RNA (dsRNA) activates TLR3, which triggers activation of the interferon-regulator factor (essentially IRF7) by recruiting MyD88 complex and promotes type I interferon that leads to anti-inflammatory antiviral response. RNA viral-activated endosomal TLRs-MyD88 complex, together with other cytokine- and peptide-signaling pathways, indirectly induce activation of the IKK kinase complex (IKKα/β/γ-NEMO), which in turn causes activation and release of the cytosolic NF-κB complex through phosphorylation/ubiquitination and degradation of the IκB element. Viral infection promotes cell stimuli that induce TNFα, IL-1, IL-1 cytokines, and Lipopolysaccharides (LPS) bacterial ligands (in secondary bacterial infection) to bind to their specific cell receptors and induce the cytosolic signal cascades towards the activation of the IKK kinase complex. Viral infections also cause intracellular stress, which disrupts cell homeostasis through mitochondrial reactive oxygen species (ROS) production, which further induces the IKK kinase complex. NF-κB complex enters the nucleus and initiates transcription of pro-inflammatory factors, including cytokines, chemokines, adhesion molecules, growth factors, and inflammatory mediators. While exacerbated production of inflammatory factors is prone to cytokine storm, healthy regulation of the inflammation can be achieved using anti-inflammatory drugs (nonsteroidal anti-inflammatory drugs—NSAIDs and steroids) [83]. (Created with BioRender.com, 2024, accessed on 6 October 2024).

Dysregulation of the NF-κB pathway can lead to excessive inflammation, contributing to pathologies in viral infections, such as the ARDS observed in severe COVID-19 cases (Figure 2) [72,73]. Oxidative stress is a crucial contributor to NF-κB activation, acting as both a signaling molecule and a mediator of inflammation [74]. During viral infections, cells generate reactive oxygen species (ROS) as part of their immune response [75]. For instance, RSV infection is associated with elevated ROS levels, which can activate the NF-κB pathway through various mechanisms [76,77]. ROS can oxidize cysteine residues in signaling proteins, leading to their activation or modification. This results in enhanced phosphorylation of IκB proteins and subsequent release of NF-κB dimers (Figure 2) [78,79]. Oxidative stress can also disrupt mitochondrial function, further amplifying inflammation [75,80,81]. The interplay between oxidative stress and NF-κB signaling is crucial: while ROS promotes inflammation to control viral replication, excessive ROS can lead to tissue damage and chronic inflammation, exacerbating respiratory conditions like asthma and COPD [82].

### 5.2. Inflammasome Activation Pathway

**Figure 3:**
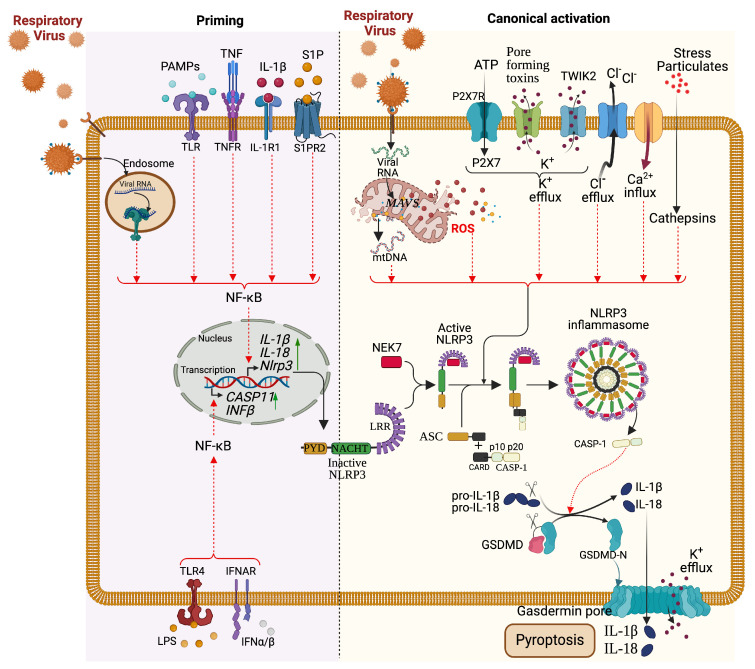
Activation of the NLRP3 Inflammasome Pathway during Respiratory Viral Infections. Upon respiratory infection, the activation of the NLRP3 inflammasome occurs generally through the canonical pathway, which includes a priming (signal 1, (**left panel**)) and an activation (signal 2, (**right panel**)) step. The priming takes place following the stress caused by the respiratory virus infection, which induces cytokines and protein ligands such as TNF, IL-1b, IFNs, lipopolysaccharide (LPS), and sphingosine-1 phosphate (S1P) to bind to their respective surface receptors. As described in Figure 2, the binding of these ligands activates their receptors and induces NF-κB activation through cytosolic signal cascades. Activated NF-κB upregulates the transcription of the Nlrp3 gene and ASC and pro-caspase1. At this stage, the respiratory viral infection, which disrupts the cell homeostasis by increasing cell stress (K+ efflux, Ca2+ flux, Cl–efflux, lysosomal disruption) and inducing mitochondrial content release [mitochondrial reactive oxygen specifies (mtROS) and oxidized mitochondrial DNA (mtDNA)], activates the NLRP3 inflammasome through oligomerization of NLRP3-NEK7, recruitment of ASC (caspase-recruitment domain), and Casp1. Auto-proteolysis of proteolytic cleavage of Casp1 releases p10/p20 active enzymes, which digest Pro-IL-1β and Pro-IL-18 into IL-1β and IL-18 cytokines to promote pro-inflammatory responses. Pyroptosis of the infected cell occurs when membrane pores are formed from digested gasdermin D (GSDMD) by the subunit p10/p20 of Casp1. (Created with BioRender.com, 2024, accessed on 6 October 2024 and adapted from [81]).

Inflammasomes are oligomeric complexes of cytoplasmic pattern recognition receptors (PRRs) that detect pathogen- and danger-associated molecular patterns, triggering inflammation crucial for immune activation. They activate inflammatory caspase-1 and induce pyroptosis, characterized by DNA fragmentation and increased membrane permeability. Respiratory virus infections disrupt redox balance through ROS and alter cytosolic ion concentrations, activating inflammasomes like NLRP1, NLRP3, and AIM2 [81,84,85]. Activated inflammasomes promote the maturation of pro-inflammatory cytokines IL-1β and IL-18, leading to leukocyte infiltration and chemotaxis. Additionally, caspase-1, released during the cascade, cleaves gasdermin D, forming pores in cell membranes and inducing pyroptosis (Figure 3) [81].

Inflammasomes, particularly the NLRP3 inflammasome, are vital for the maturation of pro-inflammatory cytokines like IL-1β and IL-18 during viral infections (Table 1). NLRP3 activation occurs in response to viral components, such as RNA and proteins, which cytoplasmic PRRs can detect [81]. For example, during infection with respiratory viruses (H1N1, RSV, and SARS-CoV-2), viral RNA triggers NLRP3 activation, leading to caspase-1 recruitment and activation. This process results in the cleavage of pro-IL-1β into its active form, promoting the recruitment of immune cells to the infection site and driving inflammation [86]. Additionally, the activation of inflammasomes can lead to pyroptosis, a form of programmed cell death characterized by cell lysis and release of inflammatory cytokines (Figure 3) [81]. While inflammasome activation is essential for effective immune responses, excessive activation can result in tissue damage and contribute to severe inflammatory conditions, as seen in acute respiratory infections [72]. However, the specific regulation of the inflammasome activation remains unclear in several RVIs (outside SARS-CoV-2, influenza, RSV).

### 5.3. Pyroptosis

Pyroptosis is a form of inflammatory cell death that serves a dual purpose during viral infections, facilitating pathogen clearance and potentially leading to tissue damage [70]. Characterized by cell swelling, lysis, and release of inflammatory mediators, pyroptosis is primarily mediated by activating inflammasomes such as NLRP3. During infections with viruses like influenza and SARS-CoV-2, the host’s immune response can lead to pyroptosis in infected epithelial cells, releasing cytokines like IL-1β and IL-18, which further recruit immune cells and intensify inflammation (Figure 3) (Table 1) [87,88]. However, excessive pyroptosis can contribute to lung injury and acute respiratory distress syndrome (ARDS) [72,73]. For instance, studies have demonstrated that excessive pyroptotic cell death in the lungs during COVID-19 correlates with severe disease outcomes [59,89,90], emphasizing the need for a balanced immune response to prevent pathological inflammation while still controlling viral replication. However, the specific regulation of pyroptosis regulation remains unclear in several RVIs (outside SARS-CoV-2, influenza).

### 5.4. MAPK Pathways

The mitogen-activated protein kinase (MAPK) pathway is another critical mediator cascade of inflammation in viral respiratory infections by translating various extracellular signals into diverse cellular responses, including proliferation, differentiation, antiviral immune response, and cell death. There are four well-studied subgroups of MAPK pathways, which include extracellular signal-regulated kinases (ERKs), p38 MAPK, c-jun N-terminal kinase (JNK/SAPK), and ERK5/Big MAP kinase 1 (BMK1) pathways. However, in humans and animals, three significant pathways (including MAPK/ERK, SAPK/JNK, and p38 MAPK) are recognized (summarized in [71]). ERK, JNK, and p38 MAPK pathways are activated upon viral recognition, facilitating the transcription of various pro-inflammatory genes [41,71,91,92]. For instance, during an influenza virus infection, the p38 MAPK pathway is activated by viral RNA, leading to increased expression of cytokines such as IL-6 and IL-8. JNK signaling is particularly important for expressing interferon-stimulated genes, which enhances the antiviral response. Moreover, MAPKs can interact with NF-κb, providing a crosstalk mechanism that amplifies the inflammatory response [71,93,94]. Dysregulation of the MAPK pathway can lead to excessive inflammation, contributing to complications like pneumonia and acute lung injury. Inhibitors of MAPK signaling have shown promise in preclinical studies for reducing inflammation and improving outcomes in models of viral respiratory infections [95,96]. However, the specific regulation of pyroptosis regulation remains unclear in several RVIs (outside SARS-CoV-2, influenza).

### 5.5. PI3K/AKT/mTOR Pathway

**Figure 4:**
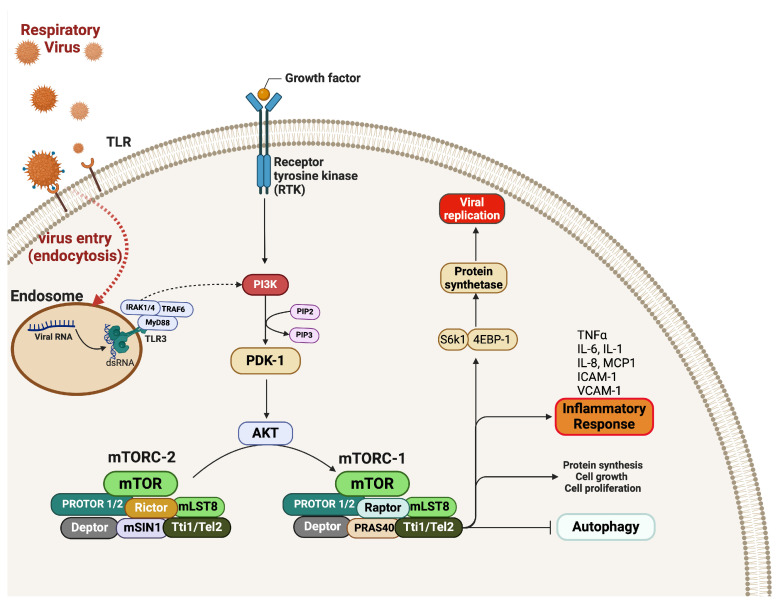
Activation of the PI3K/AKT/mTOR Pathway during Respiratory Viral Infections. The virus’s entry into the host cell leads to excessive activation of PI3K, which activates mTOR through the phosphorylation of AKT. This process ultimately enhances protein synthesis (including viral proteins), increases the production of inflammatory cytokines, and promotes the survival of infected cells. mTOR, mammalian target of rapamycin; PI3K, phosphoinositide-3-kinase; PKB (or AKT), protein kinase B; PDK1, Phosphoinositide-Dependent Kinase-1; mTORC1, mTOR Complex 1. (Adapted from [99] and created with BioRender.com, 2024, accessed on 6 October 2024).

The PI3K/AKT/mTOR pathway is one of the essential cellular signaling pathways that mediate the increase in pro-inflammatory cytokine production in response to viral infections. This pathway modulates immune responses and produces IL-10 (Table 1). However, the role of the PI3K/AKT/mTOR pathway in nasopharyngeal viral infection has not been thoroughly characterized. A study of hPIV and RSV indicates that the activation of the PI3K/AKT pathway enhances the production of pro-inflammatory cytokines and induces oxidative stress in response to hPIV and RSV infections. RSV and hPIV induced PI3K/AKT/mTOR activation in airway epithelial cells. Similarly, the severity of influenza virus infection has been associated with activating this pathway. Furthermore, a significant increase in viral titers was observed upon the inoculation of epithelial cells with the virus in the presence of PI3K inhibitors or AKT interference [97,98,99], which indicates that the virus exploits the PI3K/AKT/mTOR pathway for its fitness advantage (Figure 4). The prolonged activation of the PI3K/AKT/mTOR pathway is associated with the generation of excessive amounts of pro-inflammatory cytokines and toxicity in the lung tissue that manifests as decreased trans-epithelial electrical resistance values [100]. Because the pathway promotes cell survival and can inhibit apoptosis in infected cells, this mechanism can allow infected cells to persist longer. This could lead to chronic infection and inflammation, and contribute to the pathology of the disease. Thus, the inhibition of this pathway may enhance the antiviral response, reduce the severity of illness, and restore immune function in patients suffering from severe respiratory viral infections.

The prolonged activation of the PI3K/AKT/mTOR pathway is associated with the generation of excessive amounts of pro-inflammatory cytokines and toxicity in the lung tissue that manifests as decreased trans-epithelial electrical resistance values [100]. Because the pathway promotes cell survival and can inhibit apoptosis in infected cells, this mechanism can allow infected cells to persist longer. This could potentially lead to chronic infection and inflammation and contribute to the pathology of the disease. Thus, inhibition of this pathway may enhance the antiviral response, reduce the severity of illness, and restore immune function in patients suffering from severe respiratory viral infections.

### 5.6. Apoptosis and Inflammation

Apoptosis, typically a non-inflammatory form of cell death, can induce inflammation when cells infected by viruses release intracellular contents upon lysis. In RVIs, this phenomenon can have significant implications. For example, during adenovirus infections, apoptotic cells can release damage-associated molecular patterns (DAMPs) such as HMGB1, which activate innate immune responses. This release can enhance the recruitment and activation of immune cells, including macrophages and neutrophils, resulting in an inflammatory response [101,102]. However, if apoptosis occurs when the immune response is already heightened (such as during a severe influenza infection), it can exacerbate tissue inflammation and damage [72,73]. The relationship between apoptosis and inflammation illustrates the complexity of the immune response during respiratory infections, where the balance between clearing infected cells and preventing excessive inflammation is crucial for recovery. However, the specific pro-inflammatory responses induced by proapoptotic signaling remain unclear in several RVIs.

### 5.7. Cellular Senescence and Inflammaging

Cellular senescence plays a significant role in chronic inflammation within the respiratory system, a phenomenon termed “inflammaging”. This process, characterized by a persistent pro-inflammatory state, is particularly relevant in aging populations, where viral infections like influenza and COVID-19 can trigger senescent cell accumulation in lung tissue [103]. Senescent cells secrete various pro-inflammatory cytokines, chemokines, and proteases, a.k.a. the senescence-associated secretory phenotype (SASP), which can disrupt local tissue homeostasis and exacerbate inflammation [103,104,105]. For instance, studies have shown that senescent immune cells in older adults can impair the adaptive immune response, leading to prolonged viral persistence and more severe disease outcomes. This pro-inflammatory environment not only hinders effective viral clearance but also increases the risk of secondary infections and complications, underscoring the need for strategies targeting senescence to improve outcomes in older patients suffering from viral respiratory infections.

## 6. Virus-Induced Inflammation in Compromised Health Conditions

### 6.1. Asthma and Respiratory Virus-Induced Inflammation

Viral respiratory tract infections are thought to contribute to the development of asthma, as several research studies have identified that inflammation induced by RVIs is involved in the pathogenesis of asthma. Specifically, reports indicate that in people, especially children diagnosed with asthma, infections with RSV, hPIVs, CoVs, HRV, human bocavirus, and hMPV contribute to worsening the asthmatic phenotype (bronchiolitis) and can lead to severe exacerbations of airway obstruction, often necessitating intensive care unit admission [106,107,108]. Thus, at early ages, infections of those viruses are considered a risk factor for asthma, despite genetic factors [106,108,109]. For instance, by inducing inflammation in the epithelial airways, infection with respiratory viruses (RSV, IVs, and SARS-CoV-2) may leverage the immunoinflammatory mechanisms to enhance absorption of aeroallergens across the airway wall, increasing the likelihood of sensitization. RSV-specific IgE antibodies can cause mast-cell mediator release, leading to bronchospasm and eosinophil recruitment. Respiratory viruses can also use epithelium-secreted inflammation mediators (TNF-α/β, IL-1, IL-6, IL-8, MIP-1, RANTES, MCP-1, leukotrienes, and adhesion molecules) and lymphocyte activation mechanism to respectively intensify inflammation and stimulate a T-helper 2 (Th2)-like immune response in predisposed asthma patients [107,108,109]. However, the specific pro-inflammatory responses induced in asthmatics during RVIs remain unclear in several RVIs.

### 6.2. Bacterial Superinfection in Respiratory Virus-Induced Inflammation

Studies reported that respiratory viruses can potentially predispose to secondary bacterial infections, in which viral–bacterial coinfection constitutes a risk factor for inflammation exacerbation and the occurrence of chronic inflammatory diseases, including COPD [110]. Bacterial superinfection following respiratory infections has been linked to a high risk of severity of the infection and death [111,112]. Interestingly, during the COVID-19 pandemic, studies have suggested that around 50% of patients who died due to COVID-19 experienced a secondary bacterial infection [110]. In their review, Rossi et al. [112] discussed how respiratory viruses predispose individuals to bacterial infections and highlighted several mechanisms. Precisely, RVIs expose hidden bacterial receptors, increase bacterial adhesion, and disrupt tight junctions, compromising the epithelial barrier. Viruses also provide bacteria with nutrients like mucins and iron, alter immune responses, and impair bacterial defenses. Additionally, they change the airway microbiome, making the lungs more vulnerable to harmful bacteria (reviewed in [112]).

Viral infections with high cytotoxic potential, such as those caused by IVs, can damage the respiratory epithelium, facilitating bacterial adhesion. For instance, IVs express a cytotoxin (PB1-F2 protein) known to induce cell death and epithelium inflammation associated with the severity of pneumonia during coinfection (reviewed in [110]). Additionally, viruses may cause immunosuppression and trigger inflammatory responses that enhance the expression of receptors utilized by bacteria. Specifically, neuraminidase (NA) activity in influenza and parainfluenza viruses increases bacterial adherence by exposing pneumococcal receptors on host cells. Moreover, studies show that inhibiting viral NA can reduce bacterial adherence and invasion [110]. Furthermore, clinical evidence also indicates that influenza vaccination and influenza-specific treatment decrease the risk of secondary bacterial infections, improve the efficacy of antibiotics, and increase survival in individuals with a high risk for complications and mortality associated with influenza [110,113], highlighting the involvement of RVI in bacterial infection, and thus the importance of effective antiviral strategies in preventing superinfections [110]. However, the specific regulation of inflammatory responses induced by bacterial superinfections remains unclear in several RVIs (outside SARS-CoV-2, influenza).

## 7. Respiratory Virus-Associated Anti-Inflammatory Treatments

### 7.1. IL-1 Blockers

Interleukin-1 (IL-1) blockers have emerged as potential anti-inflammatory treatments for respiratory virus infections, particularly in severe COVID-19 [114,115] (Table 2). As key pro-inflammatory cytokines, IL-1α and IL-1β are central to the cytokine storm associated with uncontrolled immune responses during viral infections. Monoclonal antibodies and receptor antagonists targeting IL-1, such as anakinra and canakinumab, have been developed to mitigate this inflammatory response. Anakinra, a recombinant IL-1 receptor antagonist, has shown promise in observational studies, demonstrating potential survival benefits and reducing the need for mechanical ventilation among patients with severe COVID-19 (reviewed in [116] Table 2). Similarly, canakinumab has been associated with improved clinical outcomes, such as rapid restoration of oxygen levels and decreased mechanical ventilation requirements [117]. However, recent meta-analyses of randomized controlled trials indicate that while IL-1 blockade may not significantly reduce overall mortality, it appears safe and may decrease the necessity for mechanical ventilation [116]. Thus, while the clinical efficacy of IL-1 blockers in severe respiratory virus infections remains debated, they are generally well-tolerated, suggesting a need for further investigation to clarify their role in managing hyperinflammation during viral illnesses.

### 7.2. IL-6 Blockers

IL-6 blockers have emerged as potential treatments in managing the excessive inflammation often seen in severe respiratory viral infections, particularly COVID-19 [125] and influenza [126,138] (Table 2). IL-6 is a key cytokine linked to the cytokine storm syndrome, a severe immune response that can lead to significant tissue damage and worsening of disease. Elevated levels of IL-6 and its receptor (IL-6R) have been associated with critical COVID-19 outcomes, including respiratory failure and increased mortality (reviewed in [125,138]). Treatments targeting IL-6 pathways, such as monoclonal antibodies to IL-6 (e.g., siltuximab) or IL-6R (e.g., tocilizumab, sarilumab), as well as JAK inhibitors (e.g., baricitinib, ruxolitinib), aim to dampen this inflammatory response [125,126,138]. Although some studies indicate that IL-6 blockade may reduce mortality without increasing infection risk in moderate COVID-19 cases, its efficacy in critically ill patients remains less clear, possibly due to complex immune dynamics in advanced disease stages. Additionally, factors like hyperglycemia and hypertension appear to reduce the effectiveness of IL-6 inhibitors, highlighting the need for early intervention and careful patient monitoring. While further trials are needed to solidify IL-6 blockers’ role in respiratory viral treatment, current findings support their potential in mitigating severe inflammation and preventing progression in early cases [125,126,138].

### 7.3. Steroids/Corticosteroids

Systemic corticosteroids, especially dexamethasone, have become a standard anti-inflammatory treatment in COVID-19 due to their role in mitigating hyperinflammation seen in severe cases [139,140] (Table 2). Evidence from randomized controlled trials (RCTs) suggests that corticosteroids slightly reduce all-cause mortality in hospitalized patients with symptomatic COVID-19, particularly those requiring respiratory support [140,141]. For instance, in a meta-analysis of 11 RCTs with over 8000 participants, a reduction in mortality was observed, with a median difference of 30 fewer deaths per 1000 participants when compared to standard care [139]. While corticosteroids may increase ventilator-free days, the evidence remains low in certainty due to limitations such as variability in trial methodologies and confounding by early deaths [139]. Initially, the use of corticosteroids in viral infections was controversial, given mixed outcomes in other conditions like influenza [141]. However, due to the emergent findings on their benefits in COVID-19, guidelines from the NIH and WHO were updated in 2020 to recommend their use in COVID-19 patients who require oxygen support [140]. Nevertheless, important questions remain regarding optimal dosing, duration, and timing, and further research is needed, especially to clarify corticosteroid benefits in asymptomatic or mild cases, as well as potential risks like secondary infections.

### 7.4. Vitamin D

Vitamin D exerts significant immunomodulatory and anti-inflammatory effects, particularly in respiratory health. It enhances the production of antimicrobial peptides like cathelicidin, reducing viral load and supporting lung function [133,134]. Vitamin D regulates the immune response by diminishing cytokine secretion in Th1, Th2, and Th17 cells while increasing Treg cells, thereby improving outcomes in conditions such as bronchiolitis [134]. Sufficient vitamin D levels correlate with reduced severity of acute respiratory infections, including RSV, by attenuating inflammatory pathways involving IL-17 and other cytokines [134]. Regular supplementation lowers hospitalization rates and improves airway responses [135]. Additionally, vitamin D influences key signaling pathways, such as MAPK and NFκB, which are critical for cytokine regulation. While deficiency is linked to higher prevalence and severity of respiratory infections, its role in specific populations, like very low-birth-weight infants, remains complex and warrants further investigation. Overall, vitamin D is a promising adjunct for modulating respiratory inflammation and infection outcomes [142,143].

### 7.5. Emerging Therapeutics

Like the therapeutics mentioned above targeting specific pro-inflammatory cytokines and signaling pathways (IL-6, TNF-α, and NF-κB inhibitors), current therapeutic approaches targeting cellular senescence and inflammation present promising avenues for treating age-related and chronic inflammatory diseases. These promising therapeutics includes senolytics (agents that selectively eliminate senescent cells) and senomorphics (agents that modulate SASP), aiming to reduce inflammation and restore tissue homeostasis [137].

Recent preclinical studies highlight the efficacy of senolytics, such as dasatinib and quercetin, in reducing senescent cell burden and alleviating inflammation in models of osteoarthritis, pulmonary fibrosis, and cardiovascular disease (reviewed in [137]). Senomorphics like metformin and rapamycin have shown potential to suppress SASP and mitigate inflammation without eliminating senescent cells, offering a gentler approach [137].

Clinical trials are beginning to evaluate these therapies in humans. For example, early-phase studies of senolytics have shown improved physical function in patients with idiopathic pulmonary fibrosis and reduced markers of systemic inflammation in metabolic syndrome (reviewed in [136,137]). These emerging treatments hold potential for addressing a range of diseases driven by chronic inflammation, particularly in aging populations. However, challenges such as off-target effects, long-term safety, and patient selection need further exploration through comprehensive clinical trials. Also, their efficacy in reducing RVIs-induced inflammation needs further investigation.

## 8. Conclusions

In summary, the current array of knowledge strongly highlights the key role of inflammation in the pathogenesis of viral respiratory infections. Epidemiological evidence shows that anti-inflammatory agents can enhance survival during historical pandemics. Due to their essential contribution to antiviral immunity, anti-inflammatory therapies may not wholly inhibit the progression of RVIs. Anti-inflammatory agents such as corticosteroids are approved therapeutics in multiple clinical settings. However, further understanding of the potential benefits and risks associated with other anti-inflammatory agents and their combinations is required. Understanding the complex interplay between viral pathogens and the host immune response, and how inflammation can both contain and exacerbate the progression of viral infections, may lead to potential therapeutic targets for modulating the aberrant inflammatory responses during VRIs.

## Figures and Tables

**Table 1:** Key host inflammatory response and the target immune cell per respiratory virus. ↑ indicates increase.

Viruses	Inflammatory Pathway Regulation	Mediators	Refences
SARS-CoV-2	↑ inflammasome pathway ↑ pyroptosis ↑ NF-κB signaling ↑ MAPK signaling pathway ↑ PI3K/AKT/mTOR signaling pathway ↑ JAK-STAT signaling pathway	↑ pro-inflammatory responses in epithelial cells ↑ cytokines: IL-1β, IL-2, IL-6, IL-8, IL-10, IP-10, MCP-1, type I and III interferons (IFNα/β/λ) ↑ chemokines (CXCL8, RANTES) ↑ caspase-1, caspase-3, and caspase-11	[70]
Influenza viruses	↑ redox-mediated inflammasome pathway ↑ pyroptosis ↑ NF-κB and JAK-STAT signaling pathways ↑ PI3K/Akt/mTOR pathway ↑ MAPK pathway	↑ pro-inflammatory responses in endothelial cells ↑ cytokines: IL-1β, IL-6, IP-10, IL-8, type I/III interferons (IFNα/β/λ), tumor necrosis factor (TNF-α)	[63,70,71]
RSV	↑ inflammasome pathway ↑ NF-κB signaling ↑ Th2 and Th17 immune response ↑ pyroptosis ↑ IL-1R signaling	↑ Th17 cytokine, IL-17A ↑ Th2 cytokines (IL-2, IL-4, IL-5, IL-9, and IL-12) ↑ IL-4, IL-5, IL-13, and RANTES ↑ Type I and III interferons (IFNα/β/λ), ↑ pro-inflammatory cytokines (IL-6, IL-8, IL-12 in epithelial cells) ↑ IL-23 and TGF-β	[70]
HRV	↑ inflammasome pathway ↑ Th2 immune response	↑ IL-4, IL-5, IL-13, and RANTES	[70]
Other respiratory viruses	↑ inflammasome pathway ↑ immune and non-immune cell inflammation ↑ Th2 immune response	↑ type II interferon (IFNγ) ↑ pro-inflammatory cytokines ↑ Th2 mediators (IL-2, IL-4, IL-5, IL-9, and IL-12) ↑, and chemokines induced by myeloid cells ↑ caspase-1, → ↑ cleavage of gasdermin D (GSDMD) → ↑ regulated form of cell death called pyroptosis → DNA fragmentation and rapid plasma membrane permeability. ↑ IL-1b, IL-18 → ↑ leukocyte innate immune cell infiltration ↑ inflammasome activation, including NLRP3 (CoVs, PIVs, and IVs). In asthmatic patients infected with HRV and RSV, the activated Th2 immune response is biased and ↑ production of IL-4, IL-5, IL-13, RANTES and eotaxin and ↑ in eosinophilic infiltration.	[70]

**Table 2:** Therapeutics Targeting Inflammation and Antiviral Drugs.

Therapeutics	Target	Mechanism of Action	Supporting Evidence
**IL-1 Blockers**			
Anakinra	IL-1R	Recombinant IL-1R antagonist ↓ IL-1α and IL-1β from binding to IL-1R, blocking pro-inflammatory signaling [116,118] ↓ chemotactic pro-inflammation recruitment and chronic inflammation (ARDS) but does not suppress immune response [40]	↓ severe inflammation (cytokine storm) in COVID and Influenza cases [119,120] ↓ mortality and ↓ need for invasive ventilation [121] ↓ lung damage in mice infected with IV [121,122] ↓ RSV-associated inflammation in children [122,123]
Canakinumab	IL-1β	mAbs specifically targeting IL-1β, ↓ downstream inflammation and cytokine production [123]	Used off-label for COVID-19 with reports of improved outcomes in small trials [116,123] Its effectiveness may vary based on timing of administration and severity of inflammation [116,123] Effective against Influenza [120,124]
**IL-6 Blockers**			
Tocilizumab and Sarilumab	IL-6R	Humanized mAbs against IL-6R ↓ both soluble and membrane-bound IL-6R, thereby ↓ the IL-6 signaling pathway [125,126] ↓ IL-6-mediated systemic inflammation [127,128]	↓ mortality in moderate and severe COVID-19 patients by ↓ cytokine storms. ↓ in mortality with early intervention [125]
Siltuximab	IL-6	Anti-IL-6 mAb directly neutralizes IL-6, and ↓ its binding to IL-6R and ↑ inflammatory pathways [125]	↓ inflammatory markers, but effective on survival and progression in severe COVID-19 is variable [125]
Ruxolitinib and Baricitinib	JAK1/JAK2 (downstream of IL-6)	JAK inhibitor that ↓ downstream signaling from the IL-6/IL-6R pathway, ↓ inflammation by limiting cytokine signaling through JAK-STAT pathways [125]	↓ inflammatory cytokine release in COVID-19 cases [125] ↑ recovery time and reducing inflammation in COVID cases (combined with remdesivir) [125] Prevent severe inflammation
**Steroid/Corticosteroid**			
Dexamethasone	IL-1, IL-6, TNF-α	↓ cytokine release ↓ NF-kB pathway ↓ immune cell activity and inflammation	↓ mortality in severe cases requiring respiratory support; (RECOVERY, REMAP-CAP) [129,130] slight ↓ mortality (RCTs)
Baricitinib	JAK	↓ multiple cytokine pathways (IL-6, IFN-γ, etc.) [131]	↑ respiratory function in COVID-19 [131]
Methylprednisolone	IL-6, TNF-α	Anti-inflammatory effects ↓ cytokine production and ↓vascular permeability [131]	↑ respiratory function in COVID-19 ↓ mortality in COVID-19 [131] limited evidence for mortality benefit in influenza [131]
Hydrocortisone	Broadly targets inflammatory cytokines (e.g., IL-6, TNF-α)	General immunosuppression, reduces capillary leakage and inflammation [131,132]	limited evidence in ↓ mortality limited effect on ventilator-free days (RCTs in COVID-19 and Influenza) [131,132]
Prednisone	IL-1, IL-6, TNF-α	↓inflammation ↓ T-cell activation, ↓cytokine production [131]	Limited data in COVID-19 ↑ mortality and secondary infection risk in influenza-related ARDS [131,132]
Inhaled Corticosteroids (NSAIDs)	Local inflammation in airways (e.g., IL-6, IL-8)	↓ respiratory tract inflammation with minimal systemic effects [132]	Ongoing COVID-19 investigation (NCT04355637); in vitro studies suggest localized benefit Limited evidence in influenza [131,132]
**Vitamin D**			
Vitamin D	Cathelicidin induced by IL-1 & IL-23	↓ cytokine secretion in Th1, Th2, and Th17 subsets ↑ Treg, ↓ memory T cells and IL-17 [133,134,135]	Vitamin D supplementation ↓ hospitalization due to severe acute respiratory tract infection [133,134,135]
**Novel Therapeutics**			
Senolytics (dasatinib and quercetin)	SCAPs (PI3K/AKT)	↓ tyrosine kinase, ↓ cell proliferation and migration and ↑ apoptosis [136,137].	Combination of dasatinib and quercetin ↑ apoptosis more efficiently than either drug alone; ↓ many age-associated diseases and disorders in mice [136,137] Promising in human clinical trials
Senomorphics (metformin and rapamycin)	NF-κB, mTOR, IL-1α, p38-MAPK	↓ IKK/NF-κB activation ↓ p38MAPK ↓ senescence by ↓ SASP expression [137]	Effective in ↓ cellular senescence and SASPs and ↓ multiple age-associated dysfunctions (reviewed in [137])

NSAID, Nonsteroidal anti-inflammatory drug; ↑, activate/induce; ↓, inhibit/decrease/suppress. mAbs: Monoclonal antibodies; SCAPs, senescent cell anti-apoptotic pathways; SASP, senescence-associated secretory phenotype.

## Data Availability

All data reported in the study are described in the current manuscript and cited references.
